# Crystal structure of bis­[μ-meth­oxy(pyridin-2-yl)methano­lato-κ^3^ *N*,*O*:*O*]bis[chlorido­copper(II)]

**DOI:** 10.1107/S2056989015001310

**Published:** 2015-01-31

**Authors:** Sujirat Boonlue, Anchalee Sirikulkajorn, Kittipong Chainok

**Affiliations:** a Department of Chemistry, Faculty of Science, Naresuan University, Mueang, Phitsanulok 65000, Thailand; b Department of Physics, Faculty of Science and Technology, Thammasat University, Khlong Luang, Pathum Thani 12120, Thailand

**Keywords:** crystal structure, hydrogen bonds, copper(II), Cu⋯Cl inter­action, π–π stacking

## Abstract

The racemic title compound, [Cu_2_(C_7_H_8_NO_2_)_2_Cl_2_], is composed of dinuclear mol­ecules in which meth­oxy(pyridin-2-yl)methano­late ligands bridge two symmetry-related Cu^II^ ions. Each Cu^II^ ion is coordinated in a square-planar geometry by one Cl atom, the N and O atoms of the bidentate ligand and the bridging O atom of the centrosymmetrically related bidentate ligand. The separation between the two Cu^II^ atoms is 3.005 (1) Å. In the crystal, non-classical C—H⋯O hydrogen bonds, weak π–π stacking [centroid–centroid distance = 4.073 (1) Å] and weak electrostatic Cu⋯Cl inter­actions [3.023 (1) Å] link the dinuclear mol­ecules into chains running parallel to the *b* axis. These chains are further connected by weak C—H⋯Cl hydrogen bonds directed approximately along the *a* axis, forming a three-dimensional supra­molecular network.

## Related literature   

For related structures and applications of transition metal compounds with the meth­oxy-2-pyridyl­methano­late ligand, see: Pijper *et al.* (2010 ▸); Mondal *et al.* (2009 ▸); Drew *et al.* (2008 ▸); Wang *et al.* (2003 ▸); Guidote *et al.* (2001 ▸). 
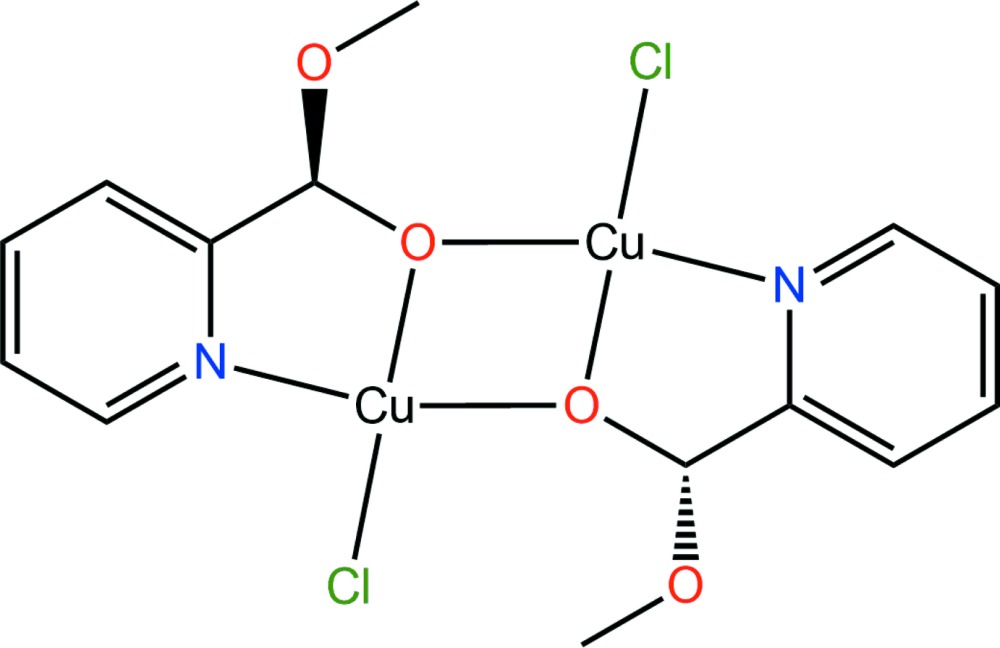


## Experimental   

### Crystal data  

[Cu_2_(C_7_H_8_NO_2_)_2_Cl_2_]
*M* *_r_* = 474.29
Monoclinic, 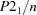

*a* = 10.5568 (14) Å
*b* = 4.0728 (6) Å
*c* = 19.257 (3) Å
β = 95.280 (3)°
*V* = 824.5 (2) Å^3^
*Z* = 2
Mo *K*α radiation
μ = 2.92 mm^−1^
*T* = 298 K
0.16 × 0.10 × 0.06 mm

### Data collection  

Bruker D8 QUEST CMOS diffractometer
Absorption correction: multi-scan (*SADABS*; Bruker, 2014 ▸) *T*_min_ = 0.645, *T*_max_ = 0.745
7703 measured reflections
1485 independent reflections
1030 reflections with *I* > 2σ(*I*)
*R*_int_ = 0.091

### Refinement  

*R*[*F* ^2^ > 2σ(*F* ^2^)] = 0.045
*wR*(*F* ^2^) = 0.108
*S* = 1.04
1485 reflections
110 parameters
H-atom parameters constrained
Δρ_max_ = 0.52 e Å^−3^
Δρ_min_ = −0.42 e Å^−3^

### 

Data collection: *APEX2* (Bruker, 2014 ▸); cell refinement: *SAINT* (Bruker, 2014 ▸); data reduction: *SAINT*; program(s) used to solve structure: *SHELXT* (Sheldrick, 2015*a* ▸); program(s) used to refine structure: *SHELXL97* (Sheldrick, 2015*b* ▸); molecular graphics: *ORTEP-3 for Windows* (Farrugia, 2012 ▸) and *DIAMOND* (Brandenburg, 2006 ▸); software used to prepare material for publication: *publCIF* (Westrip, 2010 ▸), *enCIFer* (Allen *et al.*, 2004 ▸) and *OLEX2* (Dolomanov *et al.*, 2009 ▸).

## Supplementary Material

Crystal structure: contains datablock(s) global, I. DOI: 10.1107/S2056989015001310/cq2013sup1.cif

Structure factors: contains datablock(s) I. DOI: 10.1107/S2056989015001310/cq2013Isup2.hkl

Supporting information file. DOI: 10.1107/S2056989015001310/cq2013Isup3.cdx

**Fig. 1.**
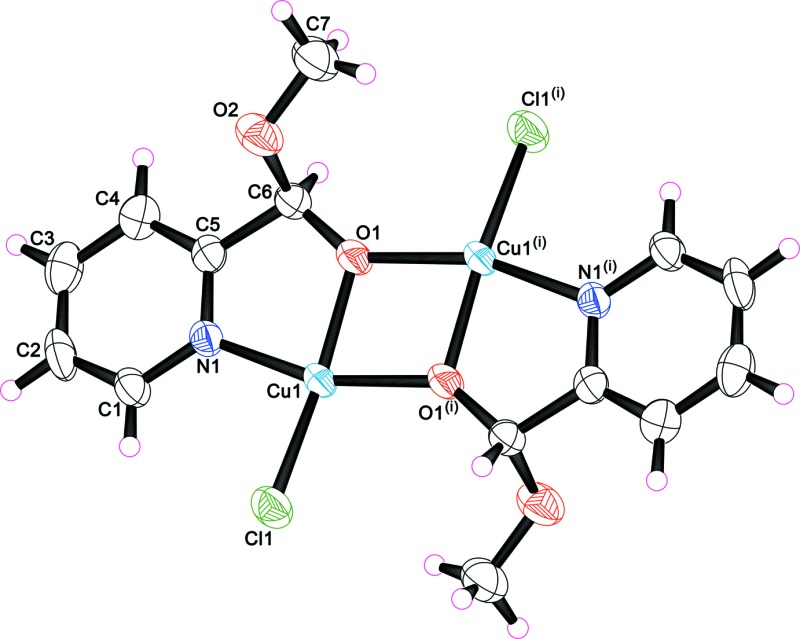
A view of the dinuclear molecule of the title compound, showing the atom labelling. Displacement ellipsoids are drawn at the 50% probability level.

**Fig. 2.**
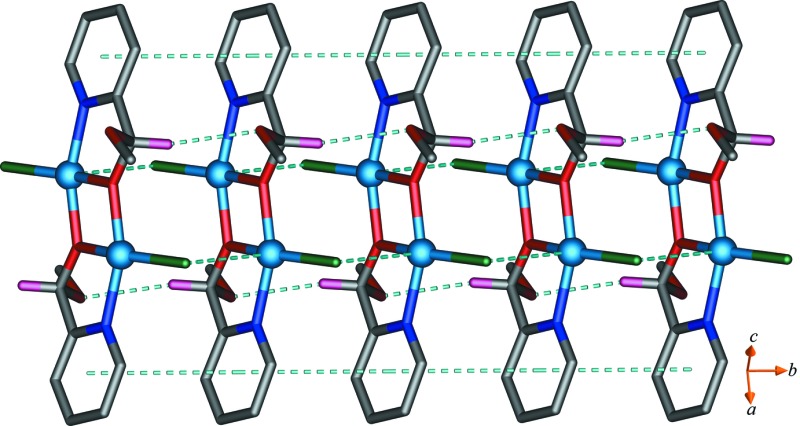
Partial packing diagram of the title compound showing a molecular one-dimensional chain running parallel to the b axis assembled from dinuclear molecules linked together through non-classical C—H···O hydrogen bonds, weak π-π stacking and weak electrostatic Cu···Cl interactions (dashed lines). Hydrogen atoms not involved in the hydrogen bonding interactions are omitted for clarity.

. DOI: 10.1107/S2056989015001310/cq2013fig1.tif
A view of the dinuclear mol­ecule of the title compound, showing the atom labelling. Displacement ellipsoids are drawn at the 50% probability level.

b . DOI: 10.1107/S2056989015001310/cq2013fig2.tif
Partial packing diagram of the title compound showing a mol­ecular one-dimensional chain running parallel to the *b* axis assembled from dinuclear mol­ecules linked together through non-classical C—H⋯O hydrogen bonds, weak π-π stacking and weak electrostatic Cu⋯Cl inter­actions (dashed lines). Hydrogen atoms not involved in the hydrogen bonding inter­actions are omitted for clarity.

**Fig. 3.**
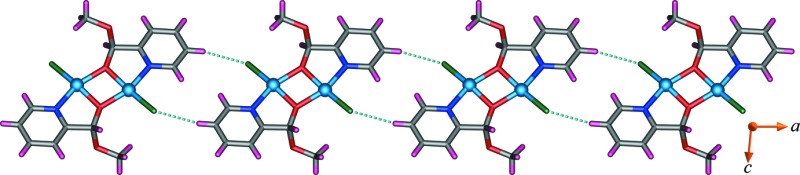
A view of the weak C—H···Cl hydrogen bonding network between adjacent dinuclear molecules in the title compound which serve to connect the chains into a three-dimensional architecture.

. DOI: 10.1107/S2056989015001310/cq2013fig3.tif
A view of the weak C—H⋯Cl hydrogen bonding network between adjacent dinuclear mol­ecules in the title compound which serve to connect the chains into a three-dimensional architecture.

CCDC reference: 1044740

Additional supporting information:  crystallographic information; 3D view; checkCIF report

## Figures and Tables

**Table 1:** Hydrogen-bond geometry (, )

*D*H*A*	*D*H	H*A*	*D* *A*	*D*H*A*
C2H2Cl1^i^	0.93	2.90	3.756(6)	154
C3H3O2^ii^	0.93	2.65	3.517(7)	156
C6H6O2^iii^	0.98	2.59	3.548(8)	165

Symmetry codes: (i) 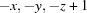; (ii) 
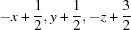
; (iii) 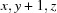.

## supplementary crystallographic information

### S1. Synthesis and crystallization

The title compound was obtained unexpectedly from the reaction of copper(I) chloride and 4-iodo-*N*-(2-pyridyl­methyl­ene)aniline in a mixed solvent of aceto­nitrile and di­chloro­methane. Typically, a solution of 4-iodo-*N*-(2-pyridyl­methyl­ene)aniline (61.6 mg, 0.2 mmol) in dry di­chloro­methane (2 ml) was placed in a test tube. A mixture of aceto­nitrile and di­chloro­methane solution (6 ml, 1:1, v/v) was carefully added on the top. A solution of CuCl (19.8 mg, 0.2 mmol) in dry aceto­nitrile (2 ml) was then carefully layered on the top of the aceto­nitrile/di­chloro­methane mixed solution. After slow diffusion at room temperature for 2 days, pale-green plate shaped crystals of the title compound were obtained.

### S2. Refinement

All H atoms were positioned geometrically and allowed to ride on their parent atoms, with d(C—H) = 0.93 Å for aromatic CH groups, 0.96 Å for non-aromatic CH groups and 0.98 Å for CH_3_ groups. The *U*_iso_ were constrained to be 1.5*U*_eq_ of the carrier atom for methy H atoms and 1.2*U*_eq_ for the remaining H atoms.

### Crystal data

**Table:**

[Cu_2_(C_7_H_8_NO_2_)_2_Cl_2_]	*F*(000) = 476
*M**_r_* = 474.29	*D*_x_ = 1.910 Mg m^−^^3^
Monoclinic, *P*2_1_/*n*	Mo *K*α radiation, λ = 0.71073 Å
*a* = 10.5568 (14) Å	Cell parameters from 546 reflections
*b* = 4.0728 (6) Å	θ = 3.6–25.4°
*c* = 19.257 (3) Å	µ = 2.92 mm^−^^1^
β = 95.280 (3)°	*T* = 298 K
*V* = 824.5 (2) Å^3^	Plate, pale-green
*Z* = 2	0.16 × 0.10 × 0.06 mm

### Data collection

**Table:**

Bruker D8 QUEST CMOS diffractometer	1485 independent reflections
Radiation source: fine-focus sealed tube	1030 reflections with *I* > 2σ(*I*)
Graphite monochromator	*R*_int_ = 0.091
Detector resolution: 0 pixels mm^-1^	θ_max_ = 25.4°, θ_min_ = 3.6°
φ and ω scans	*h* = −12→12
Absorption correction: multi-scan (*SADABS*; Bruker, 2014)	*k* = −4→4
*T*_min_ = 0.645, *T*_max_ = 0.745	*l* = −23→23
7703 measured reflections	

### Refinement

**Table:**

Refinement on *F*^2^	Primary atom site location: structure-invariant direct methods
Least-squares matrix: full	Secondary atom site location: difference Fourier map
*R*[*F*^2^ > 2σ(*F*^2^)] = 0.045	Hydrogen site location: inferred from neighbouring sites
*wR*(*F*^2^) = 0.108	H-atom parameters constrained
*S* = 1.04	*w* = 1/[σ^2^(*F*_o_^2^) + (0.0544*P*)^2^ + 0.5475*P*] where *P* = (*F*_o_^2^ + 2*F*_c_^2^)/3
1485 reflections	(Δ/σ)_max_ = 0.001
110 parameters	Δρ_max_ = 0.52 e Å^−^^3^
0 restraints	Δρ_min_ = −0.42 e Å^−^^3^
0 constraints	

### Special details

**Table:**

Experimental. SADABS-2014/4 (Bruker,2014/4) was used for absorption correction. wR2(int) was 0.0681 before and 0.0535 after correction. The Ratio of minimum to maximum transmission is 0.8650. The λ/2 correction factor is 0.00150.
Geometry. All e.s.d.'s (except the e.s.d. in the dihedral angle between two l.s. planes) are estimated using the full covariance matrix. The cell e.s.d.'s are taken into account individually in the estimation of e.s.d.'s in distances, angles and torsion angles; correlations between e.s.d.'s in cell parameters are only used when they are defined by crystal symmetry. An approximate (isotropic) treatment of cell e.s.d.'s is used for estimating e.s.d.'s involving l.s. planes.
Refinement. Refinement of *F*^2^ against all reflections. The weighted *R*-factor *wR* and goodness of fit *S* are based on *F*^2^, conventional *R*-factors *R* are based on *F*, with *F* set to zero for negative *F*^2^. The threshold expression of *F*^2^ > σ(*F*^2^) is used only for calculating *R*-factors(gt) *etc*. and is not relevant to the choice of reflections for refinement. *R*-factors based on *F*^2^ are statistically about twice as large as those based on *F*, and *R*-factors based on ALL data will be even larger.

### Fractional atomic coordinates and isotropic or equivalent isotropic displacement parameters (Å^2^)

**Table:**

	*x*	*y*	*z*	*U*_iso_*/*U*_eq_	
Cu1	0.36725 (5)	0.3633 (2)	0.49239 (3)	0.0376 (3)	
Cl1	0.25368 (12)	−0.0100 (4)	0.42980 (7)	0.0431 (4)	
O1	0.4853 (3)	0.5904 (12)	0.55882 (18)	0.0542 (13)	
O2	0.4934 (3)	0.5112 (11)	0.6792 (2)	0.0491 (11)	
N1	0.2501 (3)	0.4522 (11)	0.5649 (2)	0.0301 (11)	
C1	0.1248 (4)	0.3804 (15)	0.5620 (3)	0.0371 (14)	
H1	0.0886	0.2558	0.5248	0.044*	
C2	0.0492 (5)	0.4839 (17)	0.6114 (3)	0.0486 (17)	
H2	−0.0368	0.4299	0.6080	0.058*	
C3	0.1015 (5)	0.6680 (17)	0.6662 (3)	0.0470 (16)	
H3	0.0515	0.7428	0.7003	0.056*	
C4	0.2300 (5)	0.7412 (15)	0.6700 (3)	0.0399 (14)	
H4	0.2677	0.8658	0.7067	0.048*	
C5	0.3014 (4)	0.6271 (14)	0.6186 (2)	0.0304 (12)	
C6	0.4438 (4)	0.6921 (15)	0.6201 (3)	0.0336 (13)	
H6	0.4613	0.9267	0.6272	0.040*	
C7	0.6176 (5)	0.6071 (19)	0.7070 (3)	0.0587 (19)	
H7A	0.6792	0.5237	0.6779	0.088*	
H7B	0.6344	0.5200	0.7532	0.088*	
H7C	0.6227	0.8424	0.7086	0.088*	

### Atomic displacement parameters (Å^2^)

**Table:**

	*U*^11^	*U*^22^	*U*^33^	*U*^12^	*U*^13^	*U*^23^
Cu1	0.0240 (3)	0.0552 (5)	0.0340 (4)	−0.0123 (3)	0.0041 (2)	−0.0096 (4)
Cl1	0.0392 (7)	0.0395 (9)	0.0504 (9)	−0.0114 (7)	0.0034 (6)	−0.0065 (7)
O1	0.0265 (18)	0.099 (4)	0.038 (2)	−0.020 (2)	0.0092 (16)	−0.025 (2)
O2	0.037 (2)	0.051 (3)	0.057 (3)	−0.0029 (19)	−0.0100 (18)	0.010 (2)
N1	0.026 (2)	0.032 (3)	0.032 (2)	−0.0024 (19)	0.0013 (18)	0.006 (2)
C1	0.025 (2)	0.044 (4)	0.041 (3)	−0.002 (3)	0.000 (2)	0.007 (3)
C2	0.024 (3)	0.060 (4)	0.063 (4)	−0.003 (3)	0.013 (3)	0.014 (4)
C3	0.042 (3)	0.050 (4)	0.052 (4)	0.013 (3)	0.020 (3)	0.008 (4)
C4	0.045 (3)	0.033 (4)	0.043 (3)	0.003 (3)	0.010 (3)	−0.001 (3)
C5	0.030 (2)	0.028 (3)	0.033 (3)	0.000 (2)	0.001 (2)	0.011 (3)
C6	0.028 (2)	0.038 (4)	0.034 (3)	−0.002 (2)	0.000 (2)	0.002 (3)
C7	0.042 (3)	0.073 (5)	0.058 (4)	−0.011 (3)	−0.009 (3)	0.013 (4)

### Geometric parameters (Å, º)

**Table:**

Cu1—Cu1^i^	3.0051 (12)	C1—C2	1.365 (7)
Cu1—Cl1	2.2215 (15)	C2—H2	0.9300
Cu1—O1^i^	1.927 (3)	C2—C3	1.368 (8)
Cu1—O1	1.937 (4)	C3—H3	0.9300
Cu1—N1	1.982 (4)	C3—C4	1.385 (7)
O1—Cu1^i^	1.927 (3)	C4—H4	0.9300
O1—C6	1.361 (6)	C4—C5	1.378 (7)
O2—C6	1.415 (6)	C5—C6	1.524 (6)
O2—C7	1.424 (6)	C6—H6	0.9800
N1—C1	1.351 (6)	C7—H7A	0.9600
N1—C5	1.330 (6)	C7—H7B	0.9600
C1—H1	0.9300	C7—H7C	0.9600
			
Cl1—Cu1—Cu1^i^	139.33 (5)	C3—C2—H2	120.4
O1—Cu1—Cu1^i^	38.82 (10)	C2—C3—H3	120.6
O1^i^—Cu1—Cu1^i^	39.07 (11)	C2—C3—C4	118.8 (5)
O1^i^—Cu1—Cl1	102.14 (12)	C4—C3—H3	120.6
O1—Cu1—Cl1	165.33 (16)	C3—C4—H4	120.4
O1^i^—Cu1—O1	77.89 (16)	C5—C4—C3	119.2 (5)
O1—Cu1—N1	81.54 (15)	C5—C4—H4	120.4
O1^i^—Cu1—N1	158.20 (18)	N1—C5—C4	121.9 (5)
N1—Cu1—Cu1^i^	120.03 (12)	N1—C5—C6	116.0 (4)
N1—Cu1—Cl1	99.59 (13)	C4—C5—C6	122.1 (5)
Cu1^i^—O1—Cu1	102.11 (16)	O1—C6—O2	114.5 (5)
C6—O1—Cu1	118.6 (3)	O1—C6—C5	109.0 (4)
C6—O1—Cu1^i^	138.9 (3)	O1—C6—H6	110.2
C6—O2—C7	114.8 (4)	O2—C6—C5	102.5 (4)
C1—N1—Cu1	127.1 (4)	O2—C6—H6	110.2
C5—N1—Cu1	114.2 (3)	C5—C6—H6	110.2
C5—N1—C1	118.4 (4)	O2—C7—H7A	109.5
N1—C1—H1	118.7	O2—C7—H7B	109.5
N1—C1—C2	122.5 (5)	O2—C7—H7C	109.5
C2—C1—H1	118.7	H7A—C7—H7B	109.5
C1—C2—H2	120.4	H7A—C7—H7C	109.5
C1—C2—C3	119.1 (5)	H7B—C7—H7C	109.5
			
Cu1—O1—C6—O2	108.4 (4)	C1—N1—C5—C6	178.4 (5)
Cu1^i^—O1—C6—O2	−79.8 (7)	C1—C2—C3—C4	−0.6 (9)
Cu1—O1—C6—C5	−5.7 (6)	C2—C3—C4—C5	0.0 (9)
Cu1^i^—O1—C6—C5	166.1 (4)	C3—C4—C5—N1	1.0 (8)
Cu1—N1—C1—C2	−172.6 (4)	C3—C4—C5—C6	−178.8 (5)
Cu1—N1—C5—C4	172.9 (4)	C4—C5—C6—O1	−171.8 (5)
Cu1—N1—C5—C6	−7.4 (6)	C4—C5—C6—O2	66.5 (7)
N1—C1—C2—C3	0.2 (9)	C5—N1—C1—C2	0.8 (8)
N1—C5—C6—O1	8.4 (7)	C7—O2—C6—O1	81.5 (6)
N1—C5—C6—O2	−113.2 (5)	C7—O2—C6—C5	−160.6 (5)
C1—N1—C5—C4	−1.4 (8)		

Symmetry code: (i) −*x*+1, −*y*+1, −*z*+1.

### Hydrogen-bond geometry (Å, º)

**Table:**

*D*—H···*A*	*D*—H	H···*A*	*D*···*A*	*D*—H···*A*
C2—H2···Cl1^ii^	0.93	2.90	3.756 (6)	154
C3—H3···O2^iii^	0.93	2.65	3.517 (7)	156
C6—H6···O2^iv^	0.98	2.59	3.548 (8)	165

Symmetry codes: (ii) −*x*, −*y*, −*z*+1; (iii) −*x*+1/2, *y*+1/2, −*z*+3/2; (iv) *x*, *y*+1, *z*.
