# Supplementary figures and images for: Crystal structure of bis­[μ-meth­oxy(pyridin-2-yl)methano­lato-κ3 N,O:O]bis[chlorido­copper(II)]
Source: Acta Crystallogr E Crystallogr Commun. 2015 Jan 31;71(Pt 2):m44–5. doi: 10.1107/S2056989015001310 (PMC4384564; doi:10.1107/S2056989015001310)

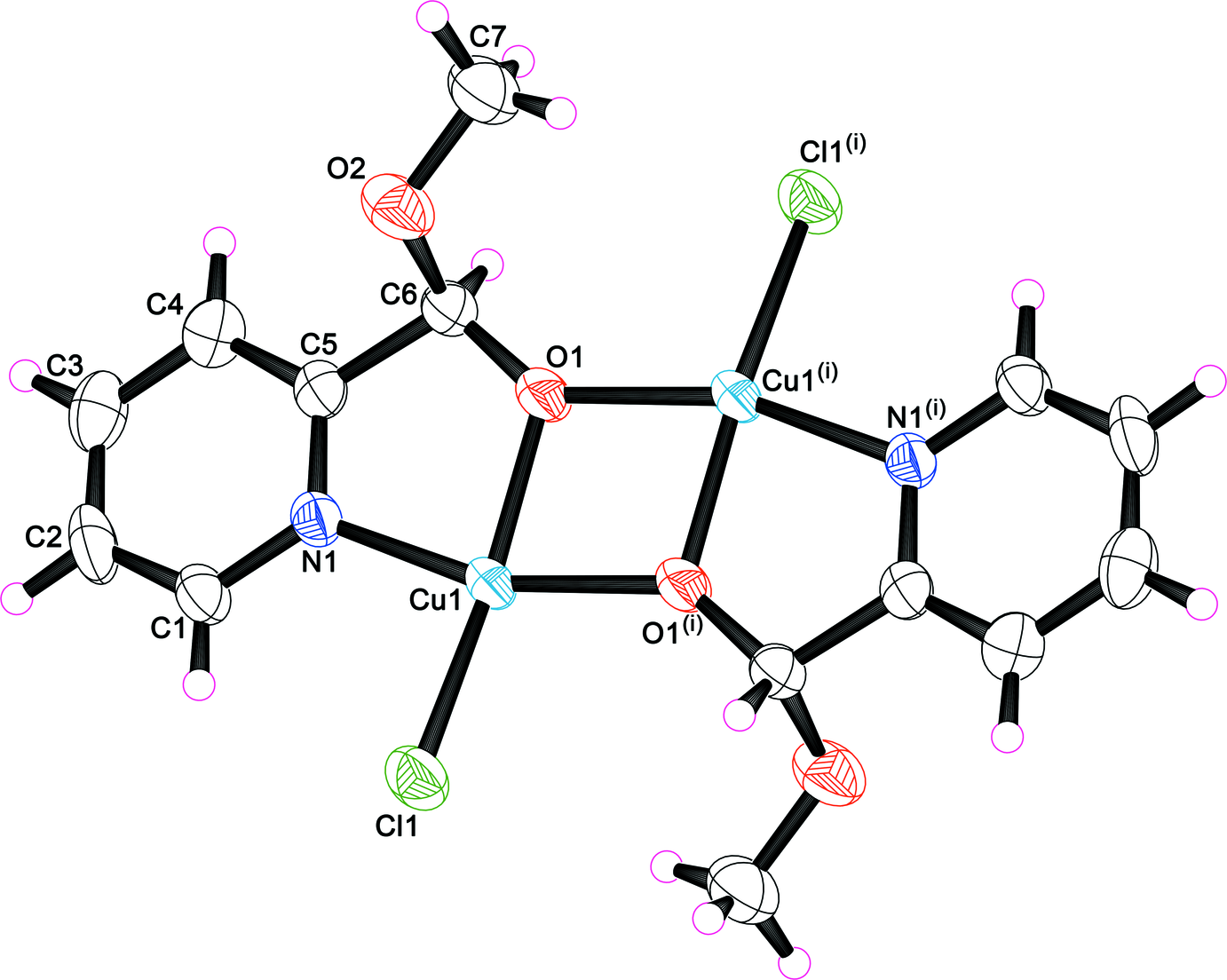

Supplement: Supplementary file 4 [file e-71-00m44-fig1.tif]

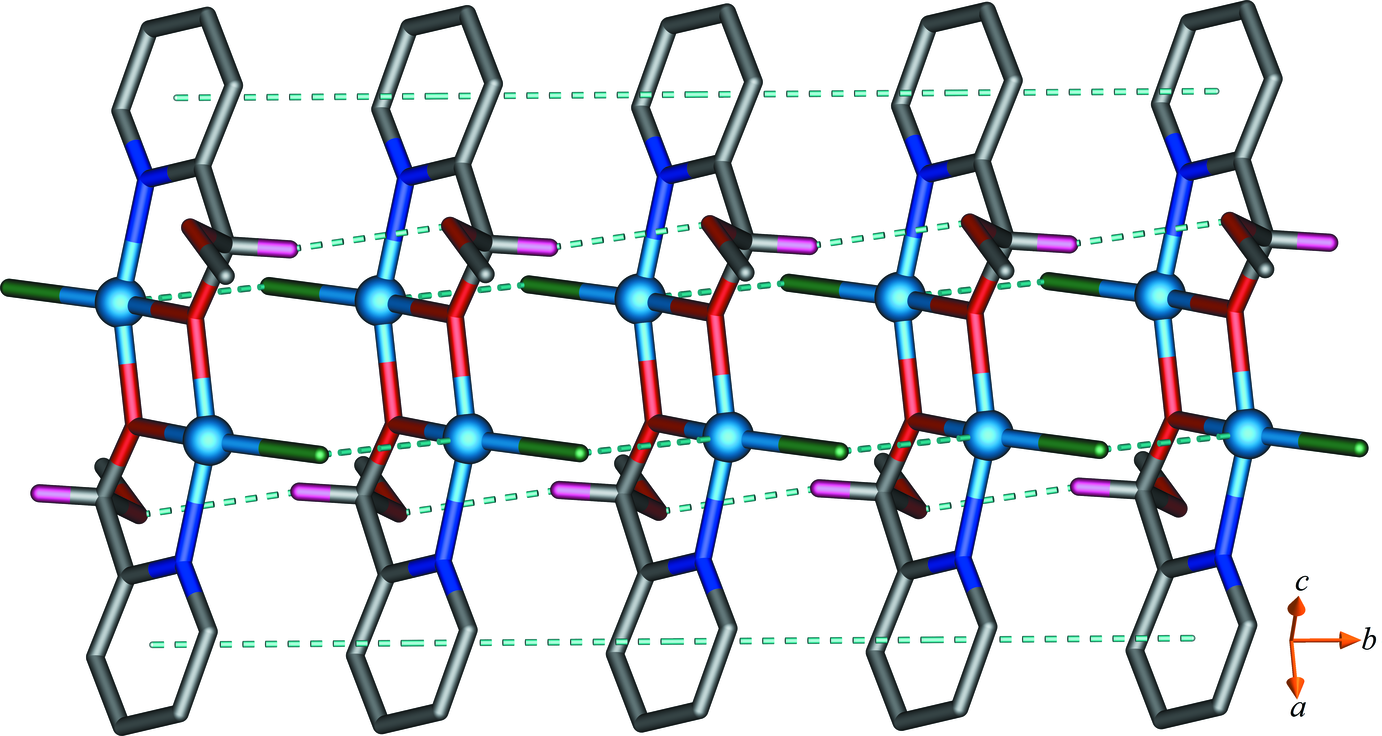

Supplement: Supplementary file 5 [file e-71-00m44-fig2.tif]

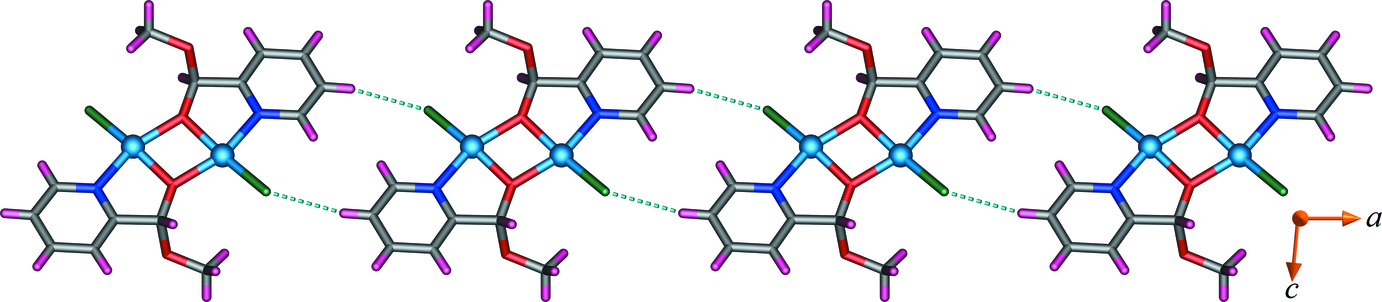

Supplement: Supplementary file 6 [file e-71-00m44-fig3.tif]
